# Monopodial and Sympodial Growth Modes in the Colonial Graptolithina (Hemichordata, Pterobranchia)

**DOI:** 10.1111/ede.70010

**Published:** 2025-06-11

**Authors:** Jörg Maletz, Rudy Lerosey‐Aubril

**Affiliations:** ^1^ Institute für Geologische Wissenschaften Freie Universität Berlin Berlin Germany; ^2^ Department of Organismic and Evolutionary Biology and Museum of Comparative Zoology Harvard University Cambridge USA

**Keywords:** coloniality, Graptolithina, monopodial growth, Pterobranchia, sympodial growth

## Abstract

Two growth modes are recognized in colonial pterobranchs (Graptolithina): monopodial growth and sympodial growth. The earliest colonial Graptolithina likely developed through monopodial growth, a mode of colony formation well‐documented in the extant graptolite *Rhabdopleura normani*. This growth involves a permanent terminal zooid and the sequential budding of additional zooids behind it, as the contractile stalk (*gymnocaulus*) of this terminal zooid elongates. This process is reflected in specific features of the secreted housing structure, the tubarium. Recently, monopodial growth was identified for the first time in a fossil taxon—the Cambrian dithecodendrid *Tarnagraptus*—based on tubarium characteristics, as no zooids were preserved. Monopodial growth also appears probable in other Cambrian taxa resembling *Tarnagraptus*, although evidence remains limited due to fragmentary materials. Sympodial growth, characterized by transient terminal zooids that are sequentially replaced as new buds form, is extensively documented in the fossil record of the Graptolithina. This growth mode characterizes the vast majority of Cambrian to Devonian Dendroidea and Graptoloidea. Phylogenetic evidence suggests sympodial growth evolved from monopodial growth in graptolithines, but the mechanisms underlying this evolutionary transition remain unclear.

## Summary

The supposedly unique monopodial clonal growth of the extant pterobranch graptolite *Rhabdopleura* is recognized for the first time in the housing construction of the Cambrian graptolite *Tarnagraptus cupidus*, linking early fossil graptolites with the extant Pterobranchia over a time distance of more than 500 million years.

## Graphical abstract

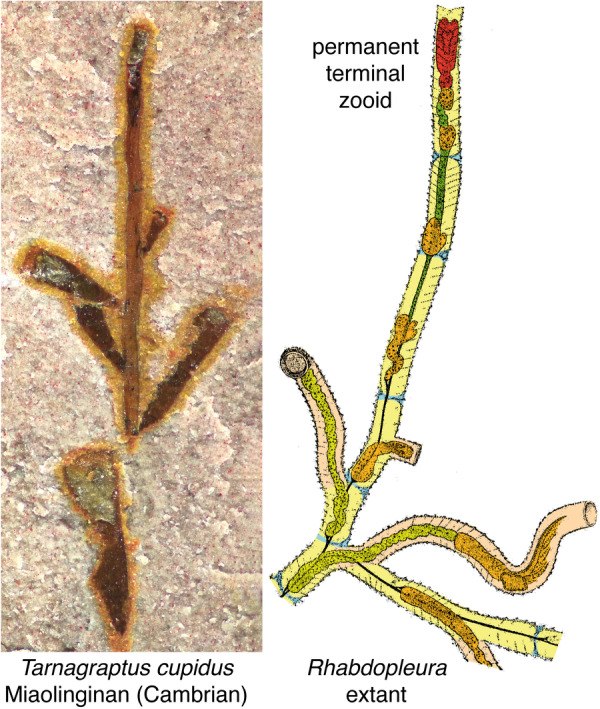

## 1 Introduction

Coloniality—agametic reproduction leading to physically connected clones—has been reported in nearly half of all animal phyla. Despite the fascination held by zoologists and philosophers since antiquity, this developmental mode and its implications remain underappreciated within theoretical ecology and evolutionary biology (Hiebert et al. 2020). Coloniality invariably occurs in aquatic, mostly marine animals and can be seen in sessile and actively or passively swimming groups, usually suspension feeders (Ryland and Warner 1986). It is well‐documented in extant and fossil Bryozoa, Cnidaria (e.g., Anthozoa, Hydrozoa and others), Hemichordata, and Porifera (but see Ereskovskii 2003). Hiebert et al. (2020, Figure 1) comprehensively reviewed the distribution of agametic reproduction in Metazoa and its relationship to coloniality; yet, many questions remain unanswered. A number of extant groups include both colonial and non‐colonial representatives, which allows the transitions or evolutionary connections between these two lifestyles to be investigated. Among the oldest examples of coloniality are fossil members of the Graptolithina. In this pterobranch subclass, like in other colonial animals, the modules (zooids) are comparatively small, measuring only 1–2 mm at most (Maletz and Gonzalez 2023), an observation that has led to hypothesize that miniaturization and anatomical modification of the zooids may accompany the acquisition of a colonial lifestyle (Ryland and Warner 1986). Support comes from the larger and more complex zooids in the exclusively non‐colonial Cephalodiscida, ranging from 1 to 10 mm in size (Maletz and Gonzalez 2023). Together with the Graptolithina, these organisms form the class Pterobranchia within the phylum Hemichordata (see Maletz 2023). The closest relatives of the Pterobranchia—the vermiform Enteropneusta—are also non‐colonial. Although the phylogeny of modern hemichordates is relatively well‐resolved, the evolutionary trajectory by which the minute colonial zooids of graptolithines have evolved from large solitary vermiform animals remains largely undocumented.

Investigating pterobranch development, especially in fossil representatives, could elucidate key aspects of colonial evolution in this group, which emerged over half a billion years ago. However, two primary challenges persist. First, extant pterobranch diversity is limited to a single colonial genus, the graptolithine *Rhabdopleura*, and two non‐colonial genera, the cephalodiscid *Atubaria* and *Cephalodiscus*. In this context, considering the substantial fossil record of the class appears critical, but this record is solely composed of fossilized remains of the tubarium, the tube‐like housing secreted by the zooids. No fossilized zooids have been convincingly documented to date (Maletz 2020, p. 45; Maletz 2024a, p. 139), with the possible exception of a single zooid from an indeterminate species of *Sphenoecium* from the Middle Cambrian of Russia, and even in this case, the available anatomical details are limited (Sennikov 2016a, 2016b).

Additionally, if paleontologists have extensively studied tubarium construction and its variation in pterobranch fossils (Bulman 1955, 1970; Maletz 2023), neontologists have mostly focused on zooid anatomy (see Gordon et al. 2023, for a rare exception). This relative disinterest for the tubarium of extant representatives is regrettable, as various aspects of the astogeny (colony development) of graptolithines can be inferred from tubarium characteristics. This review explores tubarium construction in extant and extinct pterobranchs and argues that a monopodial growth represents a plesiomorphic condition in the Graptolithina.

## 2 Coloniality in Pterobranchia

Coloniality has been documented in a variety of extant animals for a long time. Huxley (1851) already coined the term “zooid” (originally zoöid) for colonially organized tunicates, a term that was also incorporated into graptolite terminology (e.g., Bulman 1955, 1970; Hopkinson and Lapworth 1875). Kozłowski (1971) proposed to differentiate the oozooid (sexually produced individual) from the blastozooid (individual produced through asexual budding), but these terms have never gained general acceptance and “zooid” is generally used for all graptolithine zooids, including their sexually produced sicular zooid (Maletz 2023).

Graptolithina are amongst the oldest reported colonial organisms, but little is known on how this lifestyle was acquired during their evolution. Their sister group, the Cephalodiscida, did not develop a colonial lifestyle, but some representatives give hints on how coloniality might have evolved in graptolithines. In *Cephalodiscus*, the temporary colony grows through asexual budding of new zooids from the attachment disc (Maletz and Gonzalez 2023). When mature, these asexually budded zooids separate from the attachment disc of their mother zooid and secrete their own distinct organic tubes. No anatomical differences can be detected between mature sexually produced zooids and mature budded ones. Thus, the information may support the idea that the evolution of coloniality in the Pterobranchia is related to the failure of mature zooids to separate from their mother zooid. Coloniality would then be the result of a truncation of the ontogenetic development of zooids in the Rhabdopleuridae.

All graptolithines form complex entities, the colonies, in which the zooids of a colony are interconnected during their entire lives and only the founding zooid (the sicular zooid) is produced by sexual reproduction. The group includes the planktic order Graptoloidea, fossils extensively used for correlation and relative dating of Ordovician to Lower Devonian sedimentary successions worldwide. The origin of graptolithines, however, is to be found in poorly known benthic taxa, which already occur in the middle Cambrian and possibly earlier (Maletz 2024a, 2024b). The diversity of Cambrian pterobranchs is limited and their relationships with each other and with later taxa often too poorly constrained to allow assignments to known graptolithine orders (Maletz 2023; p. 188). Maletz (2024b) discussed the Miaolingian (i.e., Cambrian Series 3) record of the group, the oldest time interval represented by more than tiny fragments of tubaria. These more complete fossil materials provide us with a glimpse into how graptolite diversity and biogeographic distribution evolved during this period. The colonial organization of the tubarium is well‐documented in some of these Miaolingian taxa, but remains unknown in others. Thus, the origin of coloniality in graptolites is shrouded in the mists of the past.

Many early representatives of the Pterobranchia are rare and/or insufficiently known, owing to their preservation as fragments of tubaria flattened on sediment surfaces. More rarely, these fragments are recovered after acid dissolution of the host rock, as exemplified by rare early Cambrian (Harvey et al. 2012a, 2012b; Slater et al. 2018; Mussini and Butterfield 2025) and more abundant Lower Ordovician findings (e.g., Kozłowski 1949).

Several important features of coloniality in the Graptolithina, especially its ontogenetic and astogenetic origins, have remained virtually unstudied, even in extant representatives (species of *Rhabdopleura*). Bulman (1955, p. 20–23) introduced the terms monopodial budding and sympodial budding to discriminate two growth styles of graptolithine colonies. A sympodial mode refers to the budding of new individuals at the tip of a growing axis, whereas a monopodial mode describes the budding of new individuals from the side of a growing axis with a permanent terminal zooid. Bulman (1955, p. 23; 1958, p. 24) recognized that the growth of extant *Rhabdopleura* colonies followed a monopodial mode, which differentiated it from the sympodial development of the exclusively fossil Graptoloidea. Interestingly, monopodial growth and sympodial growth are also known in the Hydrozoa (e.g., Driesch 1890; Berking et al. 2002; Berking 2006) and is widely distributed in plants (e.g., Simpson 2006; Gerrath and Posluszny 2007).

The development of the colony in *Rhabdopleura* had already been the subject of various studies when Bulman coined the term monopodial growth to describe it. Allman (1869, p. 59, pl. 8, Figure 1) had mentioned and illustrated a ‘terminal polypide’ in *Rhabdopleura normani*, behind which buds appear and develop into normal zooids. No further details are provided in his text, but his figure, though partially inaccurate, illustrates the generation of the asexually produced zooids of the colony.

**Figure 1:**
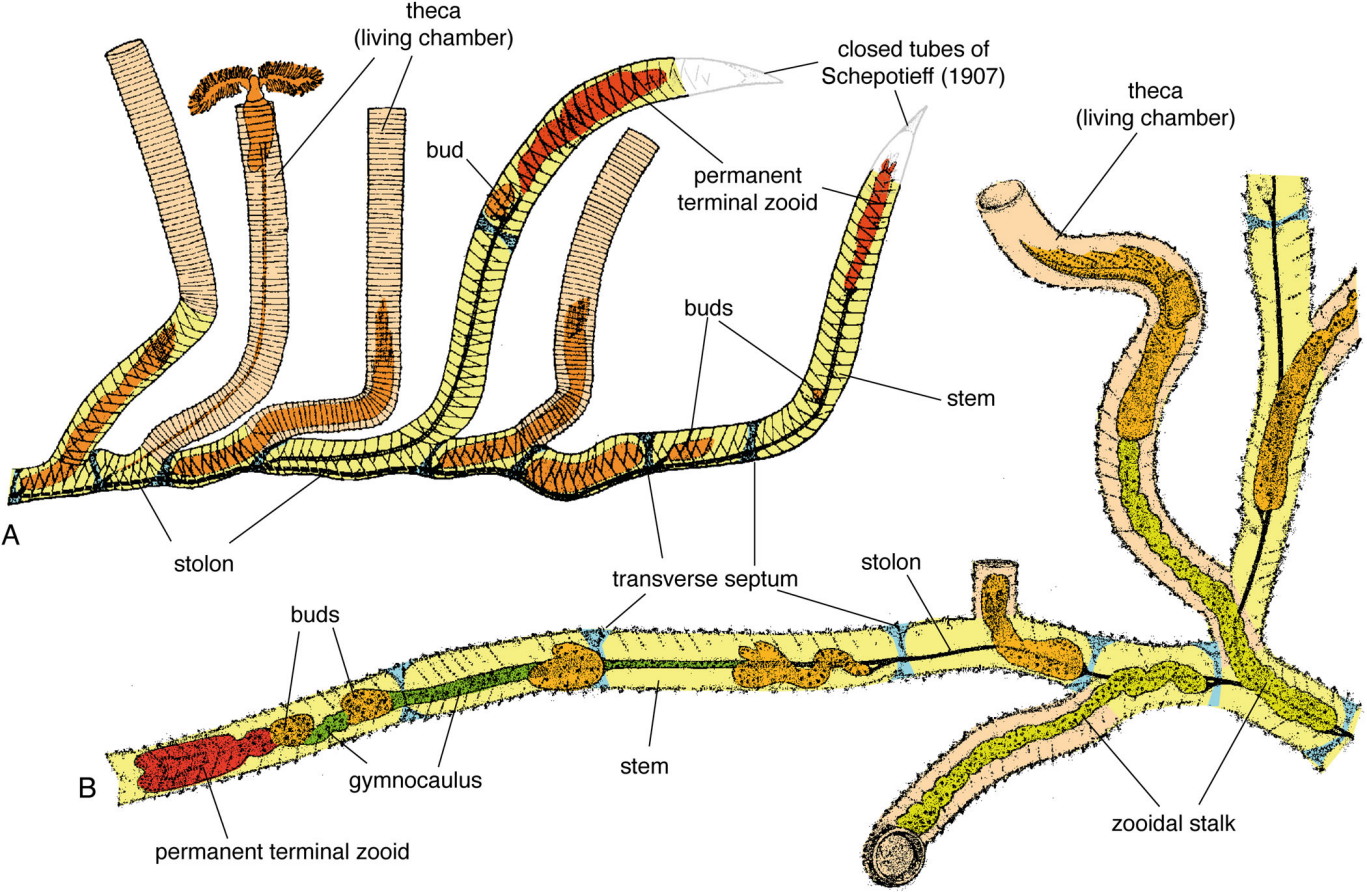
Reconstruction of growing end of a *Rhabdopleura normani* tubarium. (A) Lateral view showing two stems with permanent terminal zooids and buds behind, revised from Kozłowski (1949, Figure 11) and Maletz et al. (2016, Figure 22.1) to show terminal parts as open tubes. (B) Dorsal view, illustration after Ridewood (1907, Figure 7), based on Lankester (1884, pl. XXXIX). Stems with zigzag sutures and fusellar half rings in yellow; thecal tubes with fusellar full rings in light orange. Gymnocaulus, pectocaulus (stolon) and zooidal stalk not differentiated in (A). [Color figure can be viewed at wileyonlinelibrary.com]

Lankester (1884, pl. 39, Figure 1) (Figure 1B) identified a “gemmiferous or growing branch” and discussed the change from the gymnocaulus (unsclerotized, contractile stalk of the zooids) to the pectocaulus (the sclerotized, inflexible black stolon connecting the zooidal stalks) during the development. He distinguished “proliferous” polypides—the permanent terminal zooids in modern terminology—from “ordinary” ones (Figure 1B), based on the “curious bilobed form” of their “buccal‐discs” (Lankester 1884, p. 627). He also showed that the tubarium of the stem (his “growing branch”), with its dorsal zigzag sutures, was secreted by these “proliferous polypides” with incompletely developed arms (Lankester 1884). The term gymnocaulus has to be restricted to the part of the unsclerotized stalk of the permanent terminal zooid and the incompletely developed (immature) zooids behind it, as this stalk will eventually be sclerotized. The contractile zooidal stalk will remain flexible for the lifetime of the zooid (see also definitions in Maletz et al. (2014), thus is not homologous to the gymnocaulus (Figure 1B).

Fowler (1892, 1904) discussed and illustrated a terminal branch with a number of zooids in *Rhabdopleura normani*, but the illustrations did not add any useful anatomical details. Ridewood (1907, p. 14–15) discussed the monopodial growth, using the terminology of Lankester (1884). Schepotieff (1907, pl. 22, Figures 10–13) illustrated a permanent terminal zooid at the growing end of the axis in *Rhabdopleura* including a closed, pointed end of the tubarium, albeit without discussing it in his text. One of these figures (Schepotieff 1907, pl. 22, Figures 2, 10) (Figure 1A) was modified by Kozłowski (1949, Figure 11; same illustration in Bulman 1955, 1970) as a support to his discussion on the permanent terminal zooid. In doing so, he introduced a significant mistake in the understanding of the *Rhabdopleura* development, which appears obvious when compared to Lankester's original description.

**Figure 2:**
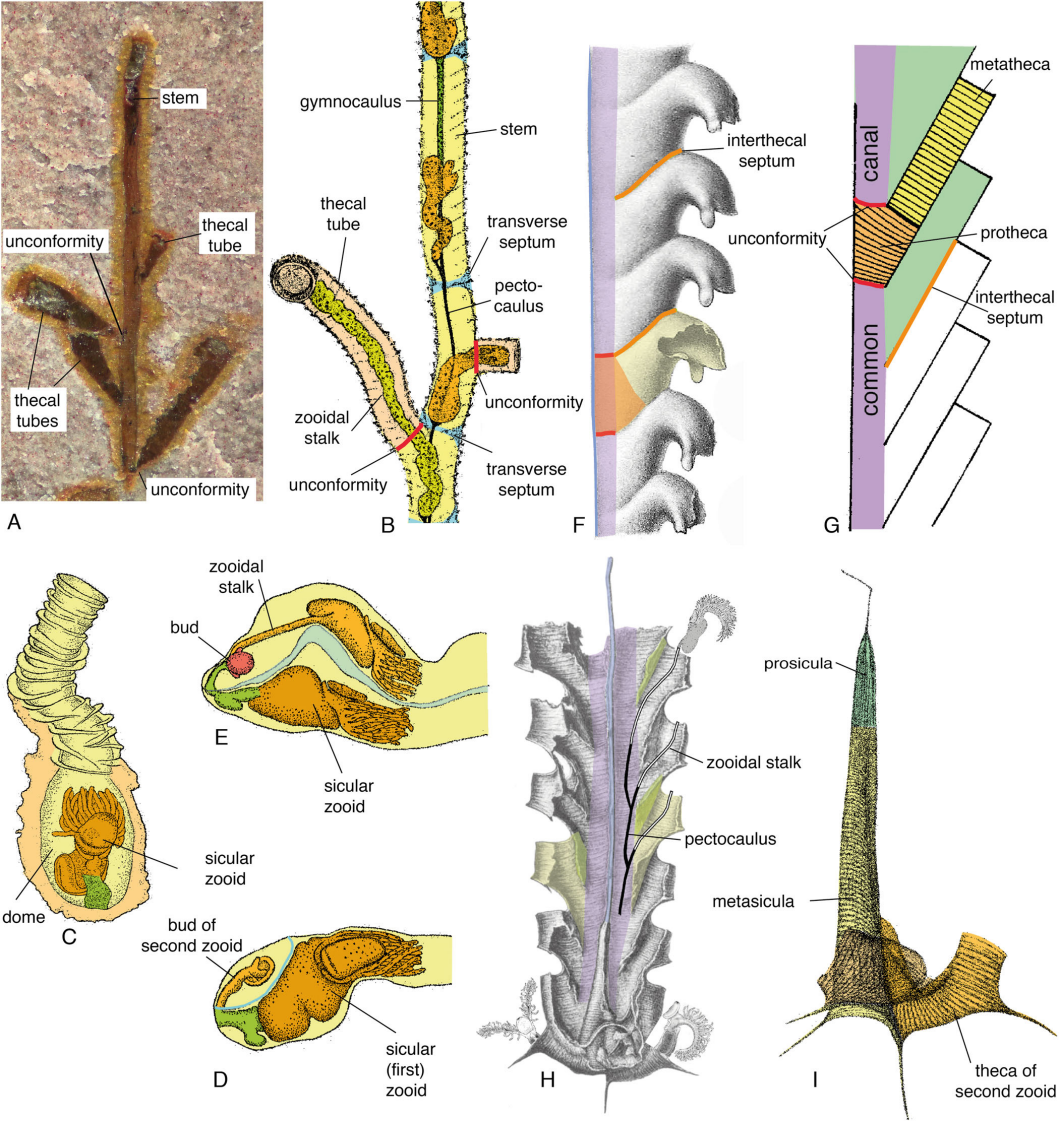
Terminology and growth of the graptolite tubarium. (A) *Tarnagraptus cupidus* Lerosey‐Aubril et al. 2024, growing end, showing monopodial growth, holotype (MCZ.IP.199849; Museum for Comparative Zoology, Invertebrate Paleontology, Harvard University, USA). (B) *Rhabdopleura normani*, growing end (see also Figure 1B). (C–E) Early astogeny of *Rhabdopleura compacta* (after Stebbing 1970). (C) Dome with sicular zooid and part of its tube. (D) Sicular zooid and immature first zooid in tubarium. (E) colony with bud (in red) of second zooid. (F–H) The common canal (in purple) in the Graptolithina, indicative of a sympodial growth. F. *Monograptus priodon*, nema seen on the left side shown in blue (after Bulman 1932a, pl. 6, Figure 1). (G) Diagram showing common canal and thecal development (after Maletz et al. 2016, Figure 15.1). (H) *Pseudamplexograptus distichus*, a biserial colony, note central nema (in blue) which is embedded in the median septum separating the two stipes, several zooids (based on *Rhabdopleura*) added, possible stolon system on right side shows pectocaulus in black, zooidal stalk in white (after Bulman 1932b, pl. 5, Figure 1). (I) *Rectograptus gracilis*, juvenile with th1^1^ complete, showing differentiation of prosicula and metasicula (after Bulman 1932a, pl. 5, Figure 12). [Color figure can be viewed at wileyonlinelibrary.com]

According to Lankester (1884, p. 636, pl. 39, Figure 1a), the growing end of the stipe (axis) is formed by an immature terminal zooid exhibiting remarkable morphological traits: a large, elongated, and distinctly bifid buccal shield (cephalic shield), rudimentary arms, and a lack of differentiation between thoracic and abdominal regions. This terminal zooid secretes the notched terminal temporal aperture from fusellar halfrings (Figure 1B, left side), and is followed by less developed zooids and buds. Schepotieff (1907, p. 215, pl. 22, Figures 10–13) interpreted the terminal zooid and the buds behind it as sterile buds and illustrated the end of the stems as closed (Figure 1A; indicated in light gray). He mentioned that these encrusting tubes differ from the normal development by the lack of a black stolon (‘Mangel des schwarzen Stolos’). The obliteration of the growing end of the stem does not make sense (Mitchell et al. 2013, p. 42) and must have been suggested by a poor preservation of Schepotieff's material. It could also be the result of a deliberate overinterpretation, as part of Schepotieff's work has proved tainted by fraud (e.g., Rimsky‐Korsakov 1911, p. 164; Tuxen 1931, p. 673; Tendal 1996, p. 82; Pass and Szucsich 2011, p. 315).

Kozłowski (1949, Figure 11) added the closed ends to the modified illustration of Schepotieff (1907, pl. 22, Figure 2) originally showing a succession of thecal tubes erupting from the stem (Figure 1A). Accepted and further discussed by Bulman (1955, 1970), this lateral budding has been regarded as the standard development of *Rhabdopleura* until now. However, an open tip of the growing end of the *Rhabdopleura* colony as described in Lankester (1884) is essential for the astogeny of the organism and an important feature for the interpretation of the astogeny of the rhabdopleurid colony (Figure 1B).

## 3 Monopodial Growth

### 3.1 Development of the Colony in Extant Rhabdopleurids

Understanding the early astogeny of *Rhabdopleura* and its tubarium may offer valuable insights into the development of the tubarium in the derived Graptolithina and clarify homology relationships within colonial pterobranchs. Stebbing (1970) provided the only available description of the early astogenetic development of *Rhabdopleura*, based on his material of *Rhabdopleura compacta*. Once mature, the sicular zooid – the primordial zooid of the colony resulting from the metamorphosis of the free‐swimming larva – attaches with its short stalk to the wall of the larval cocoon or dome (Figure 2C). The first post‐sicular zooid develops from the base of the stalk of the sicular zooid via asexual budding (Figure 2D). During the early ontogeny of this second zooid of the colony, a septum is secreted which isolates the developing zooid from the sicular zooid and creates a separate chamber, the first theca, which at that stage has no opening. When the first post‐sicular zooid is more mature, an opening is created which enables this zooid to build its own tube. At this stage, two thecal tubes grow along each other with a connecting wall (in blue in Figure 2E). A new bud is already seen at the base of the stalk of the sicular zooid (in red in Figure 2E). How the colony develops from there is unknown. The colonies of *Rhabdopleura compacta* show growth of the thecal tube in close connection to each other, forming thigmophilic (touch‐loving) colonies (Cavers 2005), which differ from the runner‐type colonies of *Rhabdopleura normani* (Maletz 2023, p. 31). Urbanek and Dilly (2000) described the stolon system of this latter species with its diaphragm complexes, but did not cover the origin of the stolon system and sicular zooid.

#### 3.1.1 Stem Tubes and Thecal Tubes

The monopodial growth of the tubarium has only been described in detail in the extant *Rhabdopleura normani* (Figure 1). This growth mode is restricted to forms with a central stem or axis (the “growing branch” of Lankester 1884, p. 625; the “Hauptröhre” of Schepotieff 1907, p. 213) from which erect thecal tubes or living chambers (the “Wohnröhren” of Schepotieff 1907, p. 213) grow by resorption of a hole into the wall of the stem. The stem is formed of fusellar half rings attached to the substrate ventrally and exhibiting a zigzag suture dorsally. The erect thecal tubes start with an unconformity on the stem wall—the result of the resorption of the original hole—and possess fusellar full rings (Figure 1). The distinction between stems and erect tubes and the different organizations of their walls were already acknowledged by Lankester (1884, p. 626–627), who regarded them as external expression of the degree of maturity of the zooids secreting them: the stems and their zigzag dorsal sutures are formed by immature‐looking zooids with bilobed cephalic shield, the erect growing tubes by mature‐looking zooids with completely formed arms. However, it should be noted that the permanent terminal zooids never develop into fully formed zooids with complete arms.

#### 3.1.2 Budding and Growth of the Tubarium

The tubarium development of *Rhabdopleura* initially starts with the dome (Figure 2C), a cocoon secreted by the founding (sicular) zooid, probably homologous to the prosicula of graptolites (Figure 2I). The dome has one resorption foramen, an opening for the emergence of the sicular zooid resulting from the metamorphosis of the larva, and a second resorption foramen for the origin of the first post‐sicular zooid (Stebbing 1970). All subsequent zooids resorb a foramen into the dorsal wall of their compartment of the growing axis.

*Rhabdopleura normani* has been regarded as the archetype of graptolites developing through monopodial growth of the colony since (at least) Ridewood (1907). As the zooids and their tubarium are available from this extant species, the development of the tubarium can be compared with the zooidal development. The axis shows the presence of a permanent terminal zooid at the tip of the stolon system (Figure 1B), behind which new buds are formed, that develop into full‐grown, mature zooids. The permanent terminal zooid shows the bilobed structure of an immature zooid as mentioned by Allman (1869) and shown by Lankester (1884) (Figure 1). It is likely that the first permanent terminal zooid of the colony is the sicular zooid, behind which the first clonally produced buds appear and grow, but additional permanent terminal zooids must be subsequently formed to produce branching colonies. Whether these new terminal zooids originate from the incomplete development of a newly formed bud (cf. Lankester 1884) or the transformation of a fully grown zooid remains undocumented, but the former hypothesis appears more likely considering the morphology of these terminal zooids. What triggers the differentiation into a terminal bud instead of a fully grown zooid is also unknown.

The buds on a given axis show a succession of growth stages with the least advanced developing bud being immediately behind the permanent terminal zooid. At the beginning of their formation, the thecal buds are not separated within the stem tube but rapidly, transverse septa appear, and each bud becomes encased into its own compartment (Figure 2B: terminal zooid not shown; see Figure 1B). Then, they continue to develop and to grow until they break through the wall of the stem tube and start secreting fusellar full rings to form their own housing tubes or living chambers, as recently documented in *Rhabdopleura emancipata* (Gordon et al. 2024, Figure 11D).

#### 3.1.3 The Stolon System: Gymnocaulus versus Pectocaulus

All graptolite colonies possess two attachment structures: the stolon and the zooidal stalk (Figures 1B and 2B). The stolon is a non‐retractable structure that extends through the stem of the colony. The still flexible stolon behind the permanent terminal zooid is identified as the gymnocaulus (Figure 2B) and is developmentally comparable with a zooidal stalk. In time it develops into the robust and inflexible ‘black stolon’ of the mature colony, or pectocaulus, as the zooids mature. A mature zooid is attached to the stolon by its zooidal stalk, a soft, retractable organ that controls the movement of the zooid in and out of its thecal tube. The zooidal stalk develops at the attachment side of the thecal bud on the main stolon during the early development of the zooid. The presence of a gymnocaulus is not verifiable in the fossil graptolites with sympodial growth, but a short and flexible gymnocaulus must be present initially, before it develops into the pectocaulus (see Figure 2H: gymnocaulus not indicated).

The development of the zooids and their connection to the stolon system is documented in detail in *Rhabdopleura normani* only (Schepotieff 1907). Urbanek and Dilly (2000) described the stolon system in *Rhabdopleura compacta*, but did not offer information on the gymnocaulus and the zooidal stalks in this species. They did not recognize a permanent terminal zooid in this species either, but according to Stebbing (1970), the sicular zooid possesses fully developed arms in this species (see Figure 2C–E). It is unclear whether the sicular zooid with its fully formed arms in *Rhabdopleura compacta* represents a permanent terminal zooid in the sense of Lankester (1884). According to Lester (1988, Figure 1), the sicular zooid of *Rhabdopleura normani* develop fully grown arms before arising from the cocoon, but the later development and the origin of the first post‐sicular zooids is unknown.

Urbanek and Dilly (2000, Figure 11) referred to the zooidal stalk in *Rhabdopleura normani* as the contractile stalk or peduncle. The most recent descriptions of *Rhabdopleura* species did not illuminate the development of these pterobranchs (Beli et al. 2018; Gordon et al. 2023).

### 3.2 Monopodial Growth in Fossils

**Figure 3:**
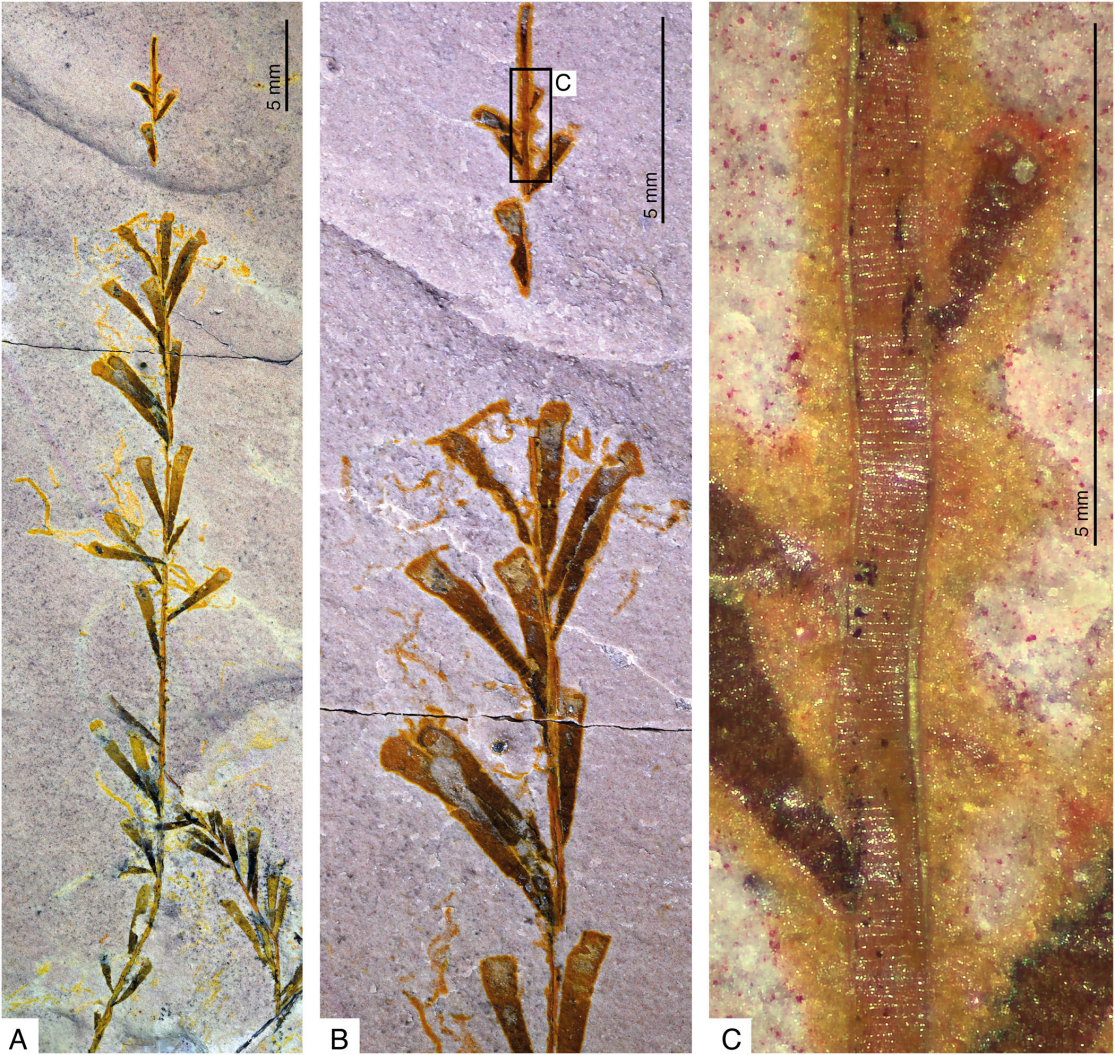
*Tarnagraptus cupidus* Lerosey‐Aubril et al. 2024 (MCZ. IP.199849, Museum for Comparative Zoology, Invertebrate Paleontology, Harvard University, USA), holotype, growing end of a larger colony, showing stem with numerous thecal tubes and monopodial development (A, B) and fusellar construction on stem (C). [Color figure can be viewed at wileyonlinelibrary.com]

Monopodial growth has not been described for fossil graptolites so far. However, the tubaria of the recently described *Tarnagraptus cupidus* (Lerosey‐Aubril et al. 2024) from the Marjum Formation of Utah, USA, can only be interpreted in this way (Figures 2A and 3). The colony shows a slender parallel‐sided stem, often composed of two or more intertwined tubes. Thecal tubes, 3–5 mm long, project from all around the stem at variable distances to each other. These thecae are characterized by their delicate nonoverlapping insertions on the stem (< 50 µm wide) and their substantial widening aperturally (up to ca. 1 mm) (Figure 3). They are distinct from the stem, from which they originate via a resorption foramen (Johnston et al. 2009). A fusellar construction of both stem and thecal tubes is documented (Lerosey‐Aubril et al. 2024), but whether half rings or full rings are present is unknown. Indeed, the superimposition of sutures from both sides of the round stems in the flattened specimens could explain the occasional observation of oblique sutures. Otherwise, the tubarium construction in this erect Cambrian taxon evocates a growth comparable to that of the encrusting *Rhabdopleura* (Figure 2A,B): the thecal tubes increase in size as their distance from the distal tip of the stem increases, as if secreted by increasingly mature zooids. In *Rhabdopleura*, this increase in size is expressed mainly as a lengthening of the parallel‐sided thecal tubes; in *Tarnagraptus*, the apex angles of the conical tubes remain approximately the same, but the length—and therefore the apertural width (Figure 3A). We regard this increase in size of the thecal tubes as evidence of increased maturity of the zooids secreting them.

A monopodial growth of the colony can be tentatively inferred for other species of *Tarnagraptus*, namely *T. cristatus, T. palma, T. robustus*, and *T. thomasi*, based on the presence of the central stem from which conical thecae originate (Figure 3; Sdzuy 1974). One fragment of *Ovetograptus gracilis* Sdzuy 1974 (SMF 30 030; Senckenberg Museum, Frankfurt collection) shows a stem with slender, parallel‐sided lateral branches that represent the thecal tubes (see Maletz 2024b, Figure 4a), a morphology suggestive of a monopodial growth. The recent reconstruction of *Ovetograptus*? sp. by Geyer et al. (2022, Figure 4) also features an axis with laterally originating thecae. A monopodial growth appears likely in *Sotograptus flexilis* Sdzuy 1974 as well. The type material of this taxon shows a robust, straight axis flanked laterally with essentially parallel‐sided thecae. Distally, the straight axis bears shorter thecal tubes (Maletz 2024b, Figure 4e), a change in size reminiscent to that observed at the tips of the stems in *Tarnagraptus* and regarded herein as indicative of a monopodial growth. The most distal thecae are interpreted as immature, not yet fully formed ones.

### 3.3 Branching

Multiramous pterobranch colonies develop through branching of the stems or stipes. In *Rhabdopleura*, branching into numerous divisions can be seen in larger colonies (e.g., Lankester 1884, pl. 37). According to Lankester (1884), this branching starts with the truncation of the developmental sequence of a zooid, but his needs to be verified. This immature zooid becomes a permanent terminal zooid, which creates a branching point in the colony via development of a new growing axis or stipe (stem in Figure 1B). Behind this permanent terminal zooid, buds develop from the gymnocaulus as the stipe grows. The genetic and molecular basis of this atypical zooid development is unknown.

## 4 Sympodial Growth

### 4.1 Development of the Colony in Graptolites

#### 4.1.1 Budding and Growth of the Colony

A sympodial mode of growth (Figure 2F–H) in pterobranchs is exclusively documented from extinct members of the Graptolithina. Its description relies on the study of the tubarium construction only and cannot be verified in extant taxa. This development produces colonies formed of a linear succession of zooids and their thecal tubes. This organization results from the fact that each newly formed zooid transiently represents the terminal zooid of the colony until it buds out to produce another zooid; in other words, there is no permanent terminal zooid (Bulman 1955, 1958; Urbanek 1986, Figure 20). This is verifiable for all taxa in which the tubarium has a common canal (Figure 2F–H), which evidence the original interconnection of the zooids via the stolon system. Another characteristic of the sympodial development is the uninterrupted transition from the proximal part (protheca) to the distal part (metatheca) of the theca (Figure 2G). The thecal tubes include part of the common canal and the fusellar halfrings show no interruption and possess a primary opening at the base of the metatheca, except for the resorption foramen for th1^1^ in the sicula. There is no secondary (resorbed) opening with an unconformity for the formation of a living chamber for the zooid, as seen in monopodial development, where thecal tubes (housing chambers) originate through resorption from the stem (Figure 2B).

#### 4.1.2 The Stolon System

The stolon system runs through a primary opening at the base of each thecal tube (Figure 2F,G). Whether this stolon system included a zooidal stalk (flexible region akin to a gymnocaulus) and a pectocaulus (non‐flexible region) as in *Rhabdopleura* cannot be ascertained, but it seems likely if the interconnected zooids were able to move in and out of their tubes.

A sympodial growth occurs in some benthic taxa and all derived planktic graptoloids (Figure 2H). However, remains of a stolon system connecting all the zooids of the colony have only been documented in a few benthic representatives, such as *Desmograptus micronematodes* (see Saunders et al. 2009). A stolon system appears also to be present in the planktic dendroid *Calyxdendrum amicabilis* Gutiérrez‐Marco and Maletz 2024. In planktic taxa, the presence of this system is inferred from the presence of a common canal running through all the thecae of the colony (Figure 2F–H). Hutt (1974) claimed that the stolon system was preserved in specimens of the anisograptid *Adelograptus tenellus* from the upper Tremadocian of England, but this was not convincingly illustrated.

### 4.2 Development of the Colony in Graptolites With Differentiated Thecae

The sympodial development was initially described from derived planktic graptoloids with a single linear succession of non‐differentiated thecae or unithecae. However, graptoloid evolution is more complex and a departure from this original organization can be seen in the Dendroidea and the Anisograptidae with differentiated thecae. At some point during the Cambrian, benthic graptolites evolved a differentiation between large autothecae and small bithecae. The origin of this morphological innovation is unknown, as the earliest diverging Graptolithina—the Rhabdopleuridae (found in fossil and extant members)—do not possess differentiated thecae, and the stratigraphic occurrence of bithecate graptoloids, which are already planktic, is limited, ranging from the top of the Furongian to the Lower Ordovician (upper Tremadocian). Differentiated thecae developed via triad budding: each mother zooid produces two morphologically distinct daughter zooids (represented by an autotheca and a bitheca), growing to either side of the mother theca (Maletz 2023). However, only one daughter zooid, the one housed in the autotheca, acts as a temporary terminal zooid and further buds into two differentiated zooids, thus ensuring the growth of the colony by distal addition of new autothecae and bithecae at the tip of the stipe (branch).

### 4.3 Branching

The branching process must have been different in extinct pterobranchs with differentiated thecae, as it involves the production of two autothecae, instead of differently sized thecae. Distinct and divergent growing axes, made of series of couplets of autothecae and bithecae, develop from these two sister autothecae (Maletz 1992). Late Tremadocian planktic graptolites do not possess bithecae anymore, but a new type of theca: the dicalycal theca. A dicalycal theca can produce two daughter thecae and thus form a branching division. Maletz et al. (2023) reviewed branching modes in fossil graptoloids, the details of which are not necessary to repeat here as a detailed comparison between graptoloids and rhabdopleurids is not possible at the moment.

### 4.4 Prothecal and Metathecal Differentiation

The tubarium of *Rhabdopleura* is differentiated into the stem and the thecal tubes (Figure 2B), while prothecae and metathecae are differentiated in the Graptolithina (Figure 2F,G). Maletz et al. (2023, p. 49, Figure 30) provided the latest understanding of their differentiation. Prothecae (Figure 2G: in orange) and metathecae (Figure 2G: in yellow) can be separated through the thecal overlap and the interthecal septum (Figure 2G). There is no unconformity visible in the formation of the fusellar half rings between protheca and metatheca and the differentiation is largely one of convenience. An unconformity exists only at the base of each protheca, where a primary opening is left for the emergence of the next zooid (Figure 2F,G: in red).

A differentiation of prothecae and metathecae is not possible in the monopodial taxa of the Rhabdopleuridae. These taxa show a main stem, separated into compartments, from which the thecal tubes grow after forming a resorption foramen and creating a new zooidal tube (Figure 2B). Thus, all thecal tubes start from a resorption foramen in the stem that is not comparable with the primary opening of thecae in derived Graptolithina featuring a common canal. A common canal simply does not exist in the Rhabdopleuridae and related taxa with monopodial growth. The stem with the compartments for the zooidal buds separated through the transverse septa (Figure 2B) cannot be identified as the homolog to the common canal of the derived graptoloids (Figure 2G). The two types of development cannot be connected constructionally, as no intermediates are currently known.

## 5 Evolution of Colonial Growth

The fossil record suggests that monopodial growth, as observed in the extant *Rhabdopleura*, is the plesiomorphic mode of development of the colony in colonial pterobranchs. This is supported by the description of monopodial growth in the middle Cambrian (Drumian) *Tarnagraptus cupidus* (Lerosey‐Aubril et al. 2024), one of the oldest examples of erect growing graptolite colonies. *Tarnagraptus* belongs to the Dithecodendridae, a group well represented in the Cambrian and likely composed of the earliest diverging members of the clade Graptolithina. Despite their morphological similarities, there is no evidence of monopodial growth in dithecodendrids other than *Tarnagraptus*, but this may be largely explained by poor preservation. These taxa, like many fossil pterobranchs, are typically known from rare fragments of flattened tubaria, which do not provide sufficient evidence to confirm the development of a stem or the differentiation of thecal types, let alone the zooidal types.

The fossil record suggests that a sympodial growth is a derived condition in the Graptolithina, which evolved in the Furongian to become a hallmark of the group. This is attested by the description of bithecate representatives dated back to this period (Callograptidae, Dendrograptidae; Maletz 2024a, Figure 7). No evidence of this developmental mode can be found in pre‐Furongian graptoloids (Maletz 2024b), and it is undocumented in the extant members of the Graptolithina.

The presence of fusellar full rings or half rings composing the tubarium is a key character in the Rhabdopleuridae, which might be used to infer the degree of maturity of the zooids that secreted different parts of the colonies in fossil taxa. In *Rhabdopleura*, the half rings of the encrusting parts are invariably formed from immature‐looking zooids and the erect growing tubes by mature and fully developed zooids. The contact with the substrate, the immaturity of the zooid, or a combination of both could explain the secretion of half rings rather than full rings. In planktic graptolites, however, contact with the substrate can be ruled out as a cause to their characteristic zigzag suture, leading to the hypothesis that their tubaria were produced by immature‐looking zooids, even though no fossilized zooids are known in these taxa. These immature‐looking zooids could be regarded as paedomorphic compared to the mature ones known in *Rhabdopleura*, and probably had evolved a distinctive morphology, as already suggested by (Melchin and DeMont 1995, Figure 1). The large conical tubes of the Dithecodendridae and Mastigograptidae would represent forms with advanced thecal growth as the sutures in their tubaria are irregular.

This hypothesis, however, is not supported by the recent description of *Rhabdopleura emancipata* (Gordon et al. 2024). This species is unique in two ways within the genus: it does not have an encrusting part of the colony and shares the erect (unattached) growth of a stem with irregularly placed thecal origins with *Tarnagraptus cupidus*. Its tubarium construction indicates that the dorsal zigzag suture found in other species of the genus does express encrusting growth: species with an encrusting tubarium part secrete half rings in an alternating fashion, species without encrusting development produce full rings. The formation of a zigzag suture and fusellar half rings is a response to environmental conditions and physical constraints in benthic taxa. Why planktic graptolites exhibit a similar tubarium construction remains unexplained.

Rigby and Sudbury (1995, p. 430) suggested the presence of three stages in the life cycle of a graptolite zooid. Only the first one, which corresponds to the secretion of the tubarium, can be studied in fossils. The second (feeding) and third (reproduction) stages remain inaccessible to paleontologists, as their potential impact on the characteristics of the tubarium is unknown. Likewise, investigating a fossilized tubarium alone does not permit to determine when new zooids of the colony were asexually produced during this life cycle. Thoroughly exploring the biology of extant rhabdopleurids, and especially this relationship between zooidal maturity and tubarium structure, could help unlock the group's extensive fossil record and, in turn, significantly enhance our understanding of the evolutionary history of these once‐thriving marine animals.

## 6 Conclusions

Monopodial growth characterizes all extant Graptolithina (*Rhabdopleura*). It is now documented in one of the oldest Cambrian dendroid graptolites and likely occurred in its similarly looking contemporaneous relatives, thus suggesting that it represents a plesiomorphic developmental trait for graptolites.

Sympodial growth is inferred in all planktic Graptolithina and in many younger, erect growing dendroids. This growth mode is reconstructed from the characteristics of the tubarium alone, as it is known exclusively from fossil taxa, and no fossilized zooids of planktic graptolites have been documented to date.

While the construction of the tubarium has been extensively studied in fossil taxa, it remains insufficiently explored in extant species.

## Conflicts of Interest

The authors declare no conflicts of interest.

## Data Availability

Data available on request from the author.
